# Heat Transfer Efficiency and pMDI Curing Behavior during Hot-Pressing Process of Tea Oil Camellia (*Camellia Oleifera* Abel.) Shell Particleboard

**DOI:** 10.3390/polym15040959

**Published:** 2023-02-15

**Authors:** Kamran Choupani Chaydarreh, Yongtao Li, Xiuyi Lin, Weiwei Zhang, Chuanshuang Hu

**Affiliations:** 1 College of Materials and Energy, South China Agricultural University, Guangzhou 510642, China; 2 College of Natural Resources and Environment, South China Agricultural University, Guangzhou 510642, China

**Keywords:** tea oil camellia, particleboard, heat transfer, sound absorption, physical and mechanical properties

## Abstract

The use of agricultural biomass composites as new construction and building materials has grown rapidly in recent decades. Considering that energy consumption is one of the most important factors in production, the aim of this work is to examine how heat transfer is affected at various ratios and combinations of three-layer tea oil camellia shell (TOCS) based particleboard with the purpose of creating a mat-forming structure, which has the best physical and mechanical properties for furniture and construction use in a dry environment and consumes the least amount of energy. Additionally, it investigated how raw materials type affects the curing process of polymeric methylene diisocyanate (pMDI) using differential scanning calorimetry (DSC). According to the obtained data, the centerline temperature could reach a maximum of 125 °C after 3 min regardless of the materials or combinations, while the pMDI curing time was 100–110 °C. The results demonstrated that efficient heat transfer could help resin polymerization and improve panel properties. The effect of raw materials on the curing behavior of resin indicated that TOCS particles somehow caused more heat reactions at the curing point. It appeared that particleboard with a ratio of 40% commercial wood particles in the surface layers and 50% TOCS particles (mesh size: −3 + 14) in the core layer with a modulus of rupture (MOR) of 11.29 N/mm^2^ and internal bonding (IB) of 0.78 N/mm^2^ has the best properties and met EN 312: 2010 standard requirements for particleboard P2.

## 1. Introduction

Currently, the great potential of wood and agriculture biomass in construction and technological innovation processes has become popular with the purpose of manufacturing particleboards [1]. Throughout the world, particleboards are used to manufacture furniture and construction materials, such as flooring systems, stair treads, and underlayment [2]. Recently, the particleboard industry has faced critical challenges regarding wood supply due to the growing global demand for raw wood materials, growing environmental concerns, and recent legislative legislation [3,4]. Hence, agricultural biomasses, with their great potential and annual access in high quantity, have been proposed as an alternative to this industry. It is a well-agreed matter that particleboard manufacturing is more sustainable than using forest residues for fuel due to environmental, economic, and social considerations. Using by-products in the particleboard industry has the potential to promote the circular economy (CE), which is a regenerative system that can balance the demand for solid wood raw materials [5]. Additionally, as one of the main principles of the CE, re-using waste raw materials and unused biomass can contribute to reducing climate change, as well as protecting the air, soils, and waters [6].

Many studies have suggested that agricultural biomasses could be used to manufacture particleboard. Despite this, each type of agricultural biomass comes with its own set of issues that prevent it from being used effectively [7]. In recent years, tea oil camellia shell (TOCS) has attracted researchers due to its great potential for being used as agricultural biomass rather than it being burned or dumped in the environment [8]. The amount of TOCS that remains useless after oil extraction from camellia fruits is estimated to be around 1.8 million tons per year in the south of China. Moreover, mainland China produced the most particleboard worldwide in 2020, amounting to 29.43 million m^3^ or 30.65% of total production. To provide wood supplies for this huge demand, TOCS might prove to be a viable alternative resource for particleboard manufacturers [9,10]. Using TOCS as an alternative material for construction materials such as particleboard aligns with the newly developed concept of green economy development. In terms of environmental and economic considerations, developing particleboard based on biomass would be advantageous [11].

However, the production of particleboard using TOCS, as with other agricultural biomasses, has to be modified in terms of their components and the types of manufacturing processes in order to attain high-quality products suitable for commercial use. In the past few years, numerous articles have been published on the improvement of particleboard properties made from different agricultural biomass. According to Laksono et al., reinforcing bagasse-based particleboard with epoxy, polystyrene, polypropylene, and phenolic can enhance its properties [12]. Regarding resin content, Zeleniuc et al. reported that increasing the adhesive content, irrespective of adhesive type, had significant effects on physical and mechanical properties [13]. To improve the properties of particleboard, Kramar et al. used basalt scrim in the center of surfaces and core layer. Despite all mentioned techniques, adding commercial wood particles to particleboard made from agricultural biomass to improve the properties is common [14]. Narciso et al. reported that commercial wood particles could be added to coconut husk-based particleboard in different ratios to enhance its mechanical properties [15].

The parameters of hot-pressing, mixing ratio with commercial wood particles, adhesive content, and particle size have been optimized in previous studies [10]. Based on the results, TOCS is highly suitable for particleboard production. However, as mentioned earlier, each new material requires extensive exploration before it can be used effectively. Particleboard manufacturing relies on hot-pressing to consolidate the mat by combining the thermal energy and the mechanical force for compression. Technically heat is transferred from press plates to the mat surface; as the temperature increases, moisture on the mat surface vaporizes and creates vapor pressure. Consequently, the vapor transfers heat from the surface layers to the center of the mat vertically. Water vaporization accelerates when the core layer reaches 100 °C, and the increased pressure causes the vapor to flow horizontally to the mat panel edges. The proper transfer of heat is crucial to sufficient resin curing, which could directly impact the strength and long-term performance of particleboard panels [16,17]. Accordingly, several parameters, such as particle geometry, density, moisture content, and temperature of press plates, influence the performance of heat transfer [18]. It is possible that sufficient heat transfer to the core layer may cause particles to become more elastic and denser, which increases the internal bonding.

This study monitored the heat behavior of TOCS-based particleboard during hot-pressing. Thus, three-layer particleboards containing TOCS particles, wood particles, and their combination were produced in different ratios. Additionally, the curing behavior of resin with different mixing ratios was studied to understand how heat transfer and resin reaction affect particleboard’s physical and mechanical properties.

## 2. Materials and Methods

### 2.1. Raw Materials

For producing the three-layer TOCS-based particleboard on the laboratory scale, TOCS was sourced from a camellia oil factory located in Guangdong province, China. The commercial wood particles, whose majority was gum (*Eucalyptus grandis* x *urophylla*) and classified in two sizes, fine (for surface layer) and coarse (for core layer), with an approximate age of five years, were sourced from a local particleboard factory in Guangdong province, China. The adhesive was polymeric methylene diisocyanate (pMDI) from Wanhua Chemical Group, Yantai, China. It has a solid content of 99.2%, a viscosity of 150–250 mPa•s, and a density of 1.22–1.25 g/cm^3^ at 25 °C.

### 2.2. Particle Preparation

After being processed through a chipper (Grinder CM200, Beijing Grinder Instrument Co., Ltd., Beijing, China), the TOCS particles were screened with ASTM standards mesh sizes of 3 (6.73 mm), 14 (1.40 mm), 20 (0.85 mm), and 30 (0.60 mm). The particles that passed mesh No.3 but remained in mesh No.14 were used for the core layer, while those that passed mesh No.20 but remained in mesh No.30 were used for the surface layers. In accordance with the previous study, these particles were of an appropriate size [19]. To achieve an approximate moisture content of 3%, all particles were oven dried.

### 2.3. TOCS-Based Particleboard Production

**Figure 1:**
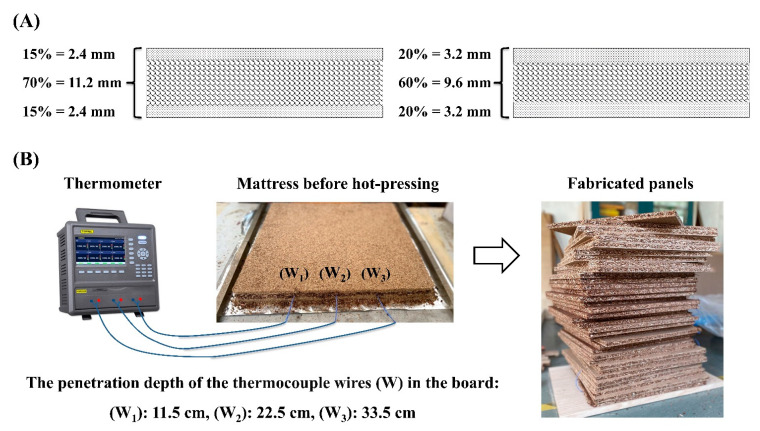
(**A**) Ratio of surface layers to core layer. (**B**) Schematic of recording heat transfer from surfaces to core layer during hot-pressing.

A computer-controlled laboratory hot press (XINXIELI Enterprise Development Co., Ltd., Suzhou, China) was used to manufacture three-layer TOCS-based particleboards with a target density of 700 kg/m^3^ (medium density particleboard (CEN/TS 16368)) [20] and a nominal thickness of 16 mm. An investigation was conducted to evaluate the effect of the ratio and combinations of core and surface layers Table 1. In a rotary lab blender, particles were resonated with pMDI adhesive (surfaces: 10%, core: 6%, based on oven dry weight), then formed into a box of 450 × 450 mm^2^ with a ratio of 15/70/15 and 20/60/20 (surface/core/surface), respectively (Figure 1).

During mat-forming, three type K thermocouples were inserted in the center of the mat to record heat transfer from the surfaces to the core layer during the hot-pressing process (Figure 1). A distance of approximately 11.5 cm was set between the thermocouples. Prior to hot-pressing, the moisture content of the mat was adjusted to 10% for surfaces and 7% for the core layer, based on the oven-dry mass of particles. The press time factor was 18 s/mm, and 3.5 MPa was considered as the picking pressure. In addition, the temperature was adjusted to 180 °C. Each treatment was replicated three times. The panels were then cut into test samples (9 samples for each test) and conditioned for two weeks in a climatic chamber at 20 °C and 65% relative humidity.

### 2.4. Characterization of Particleboards

#### 2.4.1. Mechanical Properties

The mechanical properties were measured with the help of a universal testing machine (CMT5504, Shenzhen Rethink Cooperation, Shenzhen, China). EN standards were used for mechanical tests as follows: EN 310 [21] for the modulus of rupture (MOR) and modulus of elasticity (MOE), EN 319 [22] for internal bonding (IB), and EN 13446 [23] for the withdrawal resistance of face/edge screws (FSW/ESW).

#### 2.4.2. Physical Properties

In the case of physical tests, water absorption (WA) and thickness swelling (TS) were determined using EN 317 [24], moisture content was measured through EN 322 [25], and EN 323 [26] was used for measuring the density. The vertical density profiles were measured by the X-ray densitometry DPX300-LTE IMAL-PAL Group, Russia. The compaction ratio of particleboards was calculated by Equation (1).

(1) $$\mathrm{Compaction}\;\mathrm{ratio}=\;\frac{\mathrm{PD}}{\mathrm{TD}\;\times \mathrm{TP}\%+\mathrm{WD}\;\times \mathrm{WP}\%}$$

In which PD is the density of TOCS-based particleboards; TD is the density of TOCS particles (kg/m^3^); TP% is the percentage of TOCS particles in the panel; WD is the density of wood particles (kg/m^3^); WP% is the percentage of wood particles in the panel.

A thermometer model TP700 (Shenzhen TOPRIE Electronics Co., Ltd., Shenzhen, China) was used to monitor heat transfer during the hot-pressing process.

The impedance tube AWA6128, Hangzhou Aihong instruments Co., Ltd., Hangzhou, China, was employed to measure the sound absorption, and the thermal conductivity of panels was observed with XIATECH TC3000E- Xiatech Electronics Co., Ltd., Xi’an, China.

The curing behavior of pMDI resin was studied using different scanning calorimetry (DSC) (DSC 200 F3 Maia, NETZSCH Premier Technologies, Exton, PA, USA). DSC was carried out on raw materials that had been sieved with a mesh size of NO-60 (ASTM) and had been oven dried at 103 ± 3 °C. The TOCS and wood powder were mixed with pMDI and distilled water at a 10:10:1 mass ratio, and approximately 15 g of that combination was used for each replicate. The temperature ranged from 40 °C to 200 °C at 10 °C min^−1^.

### 2.5. Statistical Analysis

One-way analysis of variance (ANOVA) was conducted in a completely randomized design and experiment with SPSS (Statistical Package for the Social Sciences) software for significant differences between factors. The *p*-value level of statistical significance was set at (*p* < 0.05). A comparison of the means was performed using the Tukey HSD test.

## 3. Results and Discussion

### 3.1. Curing Behavior of pMDI

**Figure 2:**
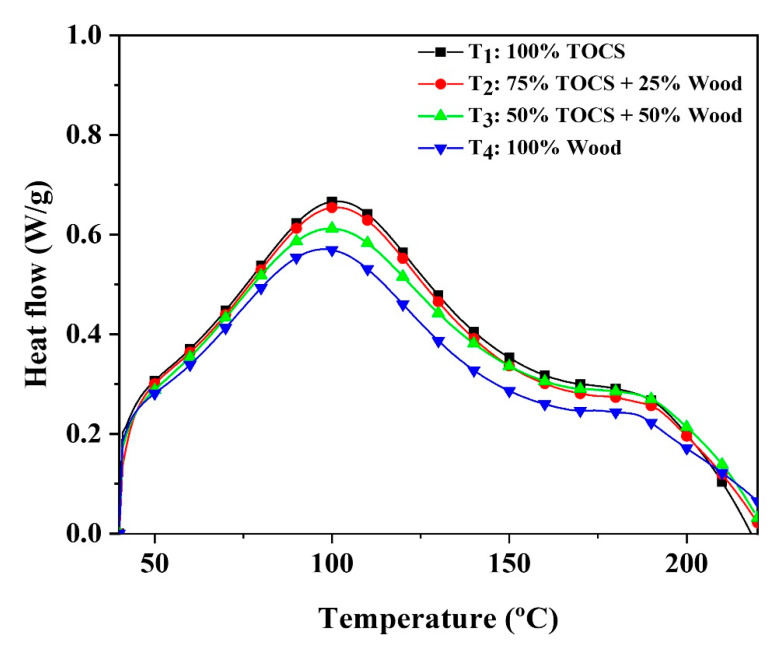
DSC curves of the curing reaction between raw materials and pMDI.

Since pMDI is free of formaldehyde, has excellent dry and wet bonding strength, high substrate moisture tolerance, and fast curing, it has a special place in wood-based panels. To understand the effects of raw materials on the pMDI curing behavior, four treatments (T_1_: 100% TOCS, T_2_: 75% TOCS + 25% wood, T_3_: 50% TOCS + 50% wood, T_4_: 100% wood) were analyzed by DSC. Three replicates were considered for each treatment. The average of obtained curves is presented in Figure 2. The results showed that the combinations with a high proportion of TOCS resulted in slightly higher reaction heat. However, the pick temperature for all treatments ranged between 100 and 110 °C, which shows the same rate of resin curing. The process of adhesion relies on mechanical interlocking and chemical reactivity to form bonds [27]. There was a possibility that the hydroxyl groups in TOCS and wood particles could react with pMDI and provide direct covalent links between the resin and particles. According to the explanations, it was expected that due to the lower amount of holocellulose in TOCS particles, which contain lower hydroxyl groups, T_1_ and T_2_ indicate lower reaction heat as compared with wood particles [9]. However, Sam-Brew and D. Smith found that hydroxyl groups in the wood had a relatively small impact on the curing behavior of pMDI, and the difference in reaction heat may cause by the chemical form bond between pMDI molecules [28]. Hence, a possible explanation for this phenomenon could be related to extractive compositions and lignin content (adhesive properties) of TOCS particles that are relatively higher than wood particles, which may cause some forms of bonding with pMDI molecules [29]. In summary, based on the other literature and the results of this study, the polymerization of pMDI is not affected significantly by the type of wood species, and in terms of cellular structure, the bonding mechanism between pMDI and wood particles is more mechanically interlocking [30]. It was found that TOCS is a viable alternative to commercial wood particles when providing inexpensive agricultural biomass that has a similar effect on resin curing.

### 3.2. Heat Transfer during Hot-Pressing Process

**Figure 3:**
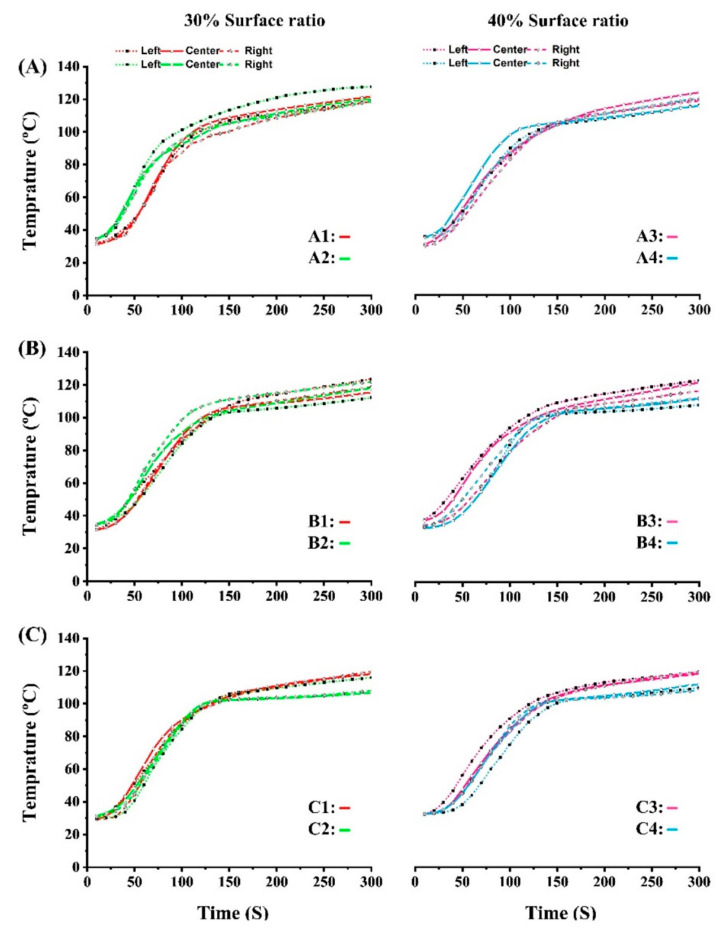
Heat transfer efficiency from surface to core layer of TOCS-based particleboards: (**A**) group A, (**B**) group B, and (**C**) group C.

The heat transfer by vapor migration from the surface of the particleboard to the center greatly influences the resin curing temperature, which may be exploited for improving certain properties of the panel [17]. Figure 3 illustrates the mean value of the heat transfer data being recorded through the surfaces of the panel to the core layer during the hot-pressing process. Three thermocouples were connected to a thermometer and placed in the center of the thickness to record the heat transfer. The maximum temperature in the particleboard’s core layer after three minutes of pressing is about 120 °C. Based on work by Solt et al., the core layer temperature is limited to 120 °C at a hot-pressing rate of 180–240 °C [31]. This temperature could provide efficient curing conditions for pMDI resin in the core layer of panels. It is known that conduction and convection are the most significant mechanisms for heat transfer into particleboard. Conduction takes place through the wood cell walls, and convection takes place through the porous spaces between the wood particles [32]. According to the findings, particleboards with a denser structure in surface layers transferred heat slower to the core layer. Due to the permeability of mats, a dense surface structure (lower porous) could be the cause of this phenomenon since a higher density could delay the rise in temperature in the panel’s interior. Furthermore, when the ratio of TOCS in the core layer was 75%, the heat was transferred to the center faster. This could be explained by the thickness of the mat, which also affects the transfer of heat, a thinner mattress was achieved by the TOCS due to the higher apparent density. Lower mat thickness could speed up the heating of the central layer since heat is transferred by regulating the flow of water vapor through the surfaces to the centerline.

### 3.3. Density, Compaction Ratio, and Moisture Content

The results of moisture content (MC), density, and compaction ratio are presented in Table 2. There is generally a relationship between the MC of particleboard and the hot-pressing parameters. According to the observed results, material combinations did not significantly affect MC. The highest MC was 9.38%, while the lowest was 8.39%. It is likely that the obtained results are caused by the adjustment of the same level of mat moisture before hot-pressing and panel density, plus the same hot-pressing process that is used to manufacture particleboards. The apparent density of particleboards varies between 695 and 712 kg/m^3^ for group A, 690 to 707 kg/m^3^ for group B, and 672 to 683 kg/m^3^ for group C. With increasing wood particles in the mat, the density of panels decreased, possibly as a result of lower apparent density of raw materials. Additionally, the bulk density of TOCS particles is higher than commercial wood particles regardless of particle size (fine or coarse). Raw materials with lower bulk density provided a higher compaction ratio, which increased the rate of spring back after production [33]. The results of the compaction ratio for group A showed the lowest ratio (0.88), while group C indicated the highest (1.04).

**Figure 4:**
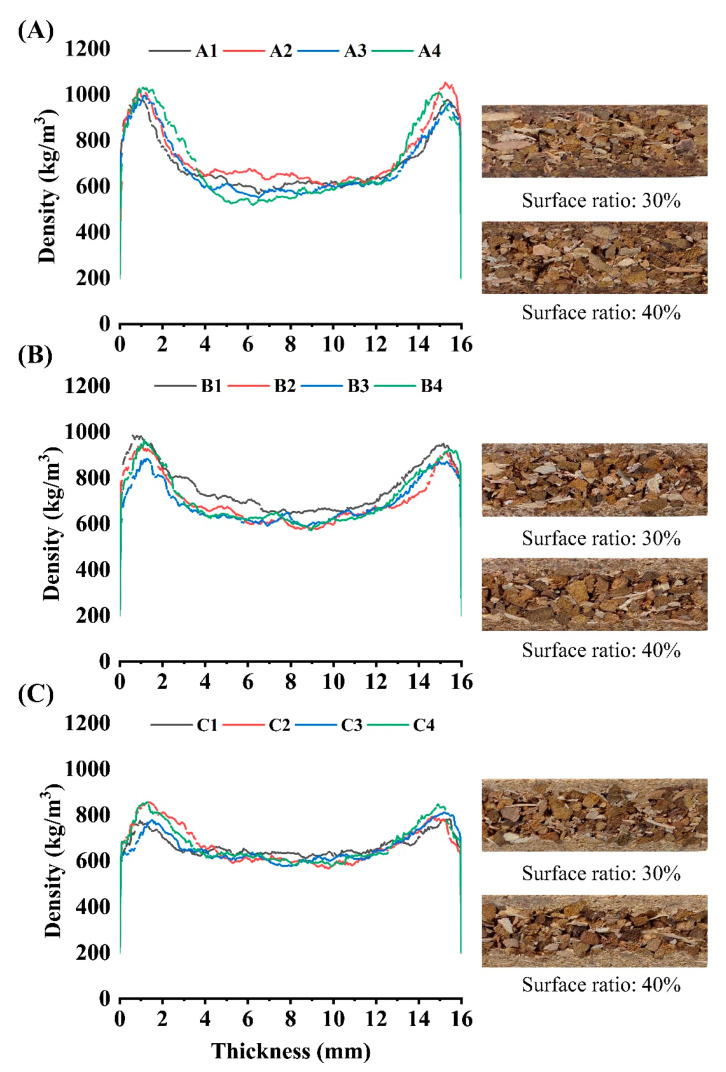
Vertical density profile of TOCS-based particleboards: (**A**) group A, (**B**) group B, and (**C**) group C.

The vertical density profile (VDP) of the specimen is presented in Figure 4. In particleboard, VDP is defined as a variation in density along the thickness centerline (geometrically symmetric) with high density at the surfaces and low density at the core layer [34]. The density distribution shows a higher pick density at first 1–2 mm from the surfaces when TOCS particles are used in the surface layers. The reason behind this trend could be related to the higher apparent density of TOCS particles (820 kg/m^3^) compared with commercial wood particles (550 kg/m^3^). Another explanation for this phenomenon is that TOCS particles used for surface layers enhanced heat transfer slightly. As shown in Figure 3, this effect was partially attributed to heat transfer. As a result of the faster rate of heat transfer from the surface to the centerline, particleboard surfaces may densify more quickly [16]. The pick density value for group A was about 1030 kg/m^3^, whereas the values for groups B and C were about 960 and 850 kg/m^3^, respectively. Conversely, group A showed a lower density in the center of the thickness, with less than 600 kg/m^3^. Because wood particles had a higher compaction ratio than TOCS particles, when commercial wood particles constituted 50% of the core layer, pick density near surfaces increased [35]. As expected, pick density distributions were wider along with the increase in surface layer ratios, while centerlines were lower.

### 3.4. Mechanical Properties

#### 3.4.1. Flexural Properties

One of the most important mechanical properties of particleboard is its bending modulus, which affects the serviceability and structural performance of both exterior and interior applications [36]. The mean values obtained for each treatment are presented in Table 3. According to BS results, panels with a higher surface layers ratio (40%) showed slightly higher values regardless of the material type. In the concept of a three-layer particleboard, most of the bear (under vertical loads) leads to the surface layers, even though it is generally acknowledged that particleboard’s flexibility depends largely on its raw material composition [5]. Hence, MOR and MOE improved with increasing commercial wood particles in the surface layers. Earlier research had shown that wood particles offered higher BS due to their higher aspect ratio and lowered apparent density [37]. In more detail, the highest MOR and MOE for group A was 6.48–1098 N/mm^2^; for group B, it was 8.97–1170 N/mm^2^; and for group C, 11.29–1415 N/mm^2^ was recorded. In terms of core layer combination, all the groups showed slightly lower MOR and MOE with 75% TOCS particles. Considering the lower aspect ratio of TOCS particles, it was determined that wood particles needed to be add to TOCS particles to achieve efficient performance. It is worth noting that in group C, which contained about 30% TOCS particles, the MOR of C4 completely met EN standard requirements for P2 type particleboard, which is 11 N/mm^2^.

#### 3.4.2. Internal Bonding Strength

The range of IB values for all treatments was between 0.71 and 0.92 N/mm^2^, higher than 0.35 N/mm^2^ defined in EN standard for P2 type particleboard. Compared to wood particles, the TOCS particles have more lignin in their chemical composition, which could enhance the particles interlocking [9]. Hence, increasing TOCS particles in the core layer provided better IB resistance. According to the trend, panels with a surface layer ratio of 30% resulted in a higher IB than panels with a surface layer ratio of 40%, suggesting that its increase could be due to more effective heat transfer that affects resin polymerization and lignin reaction (self-bonding mechanism was created by the condensation reactions of lignin) [38,39]. In addition, the particle geometry and density can significantly affect IB values. Based on the VDP graph, the specimens with higher density in the centerline resulted in better IB values.

#### 3.4.3. Screw Holding Resistance

In technical terms, both surface and core layer quality can affect FSW and ESW. Hence, it is crucial to determine the screw-holding strength in the face or edge of the particleboard. The screws are generally inserted at the centerline of the panels, where the three-layer particleboard strength is presumably the low with the most variable strength. It was demonstrated that chemical composition, apparent particle density, particle size, and core layer density might affect the ESW [40]. The specimens indicated a FSW rate of 667 to 966 N. As indicated by FSW results, this property improved by increasing the surface layer ratio from 30% to 40%. In the VDP graph above (Figure 4), a surface ratio of 40% resulted in a denser zone close to the surface, which can lead to more materials involved with the screw [41]. A study by Brito et al. showed that particleboard screw holding varies directly with particle density and particle geometry [1].

In comparison with wood particles, the geometry of TOCS particles may result in low adhesive availability per particle due to their greater specific surface area, therefore resulting in decreased screw holding. Additionally, TOCS contains less cellulose than wood particles, which has a thinner cell wall that affects the FSW/ESW of panels [3]. Hence, wood particles were added to the surface or core layers to improve these properties. The lowest ESW was recorded for A1 (501 N), whereas the highest was recorded for C4 (684 N). Interestingly, group A (100% of TOCS as surface) showed approximately similar screw-holding resistance to the other groups. According to Table 2, a linear relationship was found between the compaction ratio and ESW. As a result, a higher ESW would be possible with an increase in the compaction ratio.

### 3.5. Physical Properties

#### 3.5.1. Dimensional Stability

Regarding dimensional stability and durability, biomass-based particleboards are highly susceptible to water absorption. The results of physical properties are summarized in Table 4. Based on theory, all parameters in this study, including particle size, materials combination, and layers ratios, could influence the physical properties of boards. It should be mentioned that boards were not treated with hydrophobic agents to increase their water resistance. Regarding the results of WA, specimens had an absorption range of 42.55% to 68.95% after 24 h of immersion in distilled water. Despite group A, the core layer combination did not show any significant alteration; on the other hand, changes in the surface layer combination affected WA. In comparison to groups A (56.72–68.95%) and B (58.25–60.47%), group C (42.55–52.69%) showed lower values. It was found that increasing the wood particles in TOCS-based particleboard improved the WA properties [42]. However, some proposed substances, such as sago starch, paraffin, amino silane, and wax, could enhance physical properties.

The mean values of TS for TOCS-based particleboards ranged from 10.72% to 13.38%, somehow similar to WA; the TS of boards followed the same trend. Anatomical structures of the cell wall may contribute to this trend, as swelling is caused by an increase in water within the wall [43]. This study found that TS was lower than 14% for all treatments that conform to EN 312: 2010 type P3 for non-loadbearing applications in humid environments. Nevertheless, furniture and indoor construction requiring dry conditions are typically the most suitable intent for TOCS-based particleboard.

#### 3.5.2. Sound Absorption

**Figure 5:**
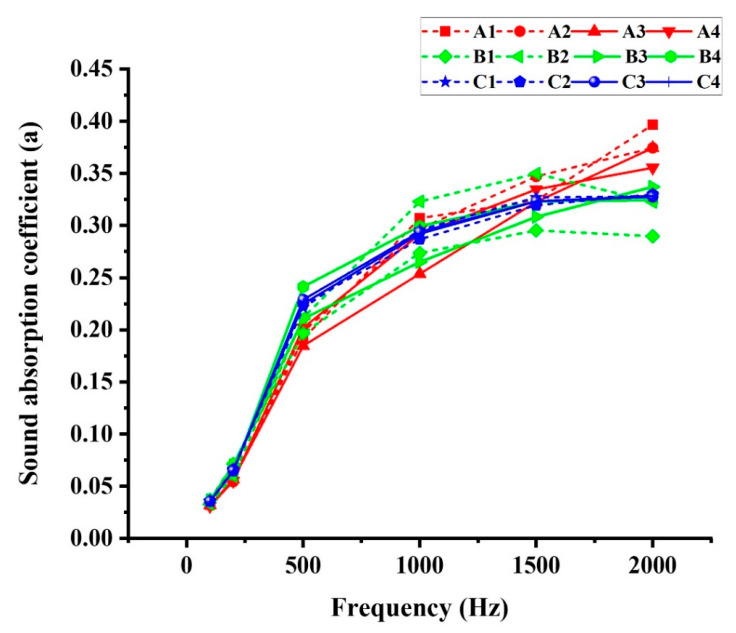
Sound absorption coefficient of TOCS-based particleboards.

In modern life, noise control has become increasingly important; hence, for insulation in buildings, composites made from agricultural biomasses are being researched since they have a low energy consumption during their manufacture and are biodegradable. The sound absorption coefficient α of TOCS-based particleboards was recorded in a frequency range of 100 to 2000 Hz and is shown in Figure 5 and Table 5. The sound absorption coefficient was dependent on the geometry and density of raw materials, which is directly affected by the porosity of panels [44]. According to Barbu et al., samples with low surface layer density and high porosity exhibited the best sound absorption properties [45]. At low frequencies between 100 and 500 Hz, all treatments followed the same trend, and there was no significant difference among the groups. However, above 1500 Hz (high frequency), group A performed slightly better in terms of absorption of sound, with a value of around 0.4. With regard to the results of VDP, group A demonstrated higher density at surface layers compared to group B and C, but the previous study showed that particleboards with 100% TOCS particles had slightly higher porosity than particleboards with 100% wood particles, despite the higher density in surface layers of group, which was most likely caused by apparent density of raw materials [37]. Additionally, panels with a 30% ratio of surface layers were found to absorb sound better due to their lower density and higher porosity [46]. According to ISO 11654 [47], the TOCS-based particleboards produced in this study were compliant with the sound absorption coefficient level rating for rooms with sound absorption classes D and C. In terms of particle geometry Ferrandez-Villena et al. reported that, in the same density, panels with bigger particles indicated better sound absorption coefficient [48]. The reason for this conclusion is that bigger particles create more empty spaces. However, this is not consistent with the results observed in this study.

#### 3.5.3. Thermal Conductivity

In recent years, agricultural biomass has gained popularity as an insulating material because of its low thermal conductivity, low environmental impact, high porosity, and low density. Buildings can benefit from these materials by managing energy efficiently and maintaining comfortable temperatures [49]. A thermal conductivity of 0.1323 to 0.1742 W/mK was observed for the TOCS-based particleboards in Table 5. Generally, the thermal conductivity of particleboards is directly influenced by densities, the higher densities result in higher thermal conductivity [50]. Thus, the thermal conductivities of group A with TOCS particles are slightly higher than others due to a higher pick density in the surface layers of particleboards in group A. In addition, it was observed that with increasing the surface layer ratio, thermal conductivity decreased, which could be explained by decreasing density close to surfaces as fine particles increased. Fine particles provide higher bulk densities, which reduce compaction and result in lower densities. According to Table 2, panels in group C had lower densities, thus indicating the best value for thermal conductivity (0.1323 to 0.1505 W/mK).

## 4. Conclusions

This study was conducted due to the consumption of materials and energy, which mainly has a significant share of particleboard production and is required for optimization. Therefore, the heat transfer efficiency of particles used for three-layer TOCS-based particleboards was investigated using different material combinations and structures. In addition, pMDI curing behavior with DSC was studied according to raw material types and combinations. The observed results indicated that heat transferred more quickly from surfaces to centerlines of boards when TOCS particles were used on surfaces, resulting in a higher IB value due to better resin polymerization. Furthermore, heat transfer on VDP was found to be effective. By transferring heat efficiently, the particles are densified properly, resulting in improved properties of boards. A higher density was recorded for the first 1–2 mm of panels surfaces with the core layer combination of 50% commercial wood particles, thus improving the BS and screw-holding resistance of panels. However, the DSC results did not indicate a significant difference between TOCS and wood particles; the reaction heat was somehow higher for TOCS particles. Due to their porous structure, it was found that using TOCS particles as surface layers resulted in a higher sound absorption coefficient. A direct correlation was found between thermal conductivity and heat transfer; a higher portion of TOCS particles led to higher values in these properties. In summary, the results show that a portion of 50% TOCS particles as a cheap raw material in the core layer could provide standard properties. Additionally, its portion in the core or surface layer showed a slight effect on heat transfer, which, on an industrial scale, can be useful for optimization energy.

## Figures and Tables

**Table 1:** The experimental design of particleboards.

Group	Treatment	Surface Ratio	Surface Layers Combination	Core Layer Combination (TOCS to Wood)
A	A1	30%	100% TOCS fine particles	75:25
	A2	30%		50:50
	A3	40%		75:25
	A4	40%		50:50
B	B1	30%	50% TOCS + 50% wood fine particles	75:25
	B2	30%		50:50
	B3	40%		75:25
	B4	40%		50:50
C	C1	30%	100% Wood fine particles	75:25
	C2	30%		50:50
	C3	40%		75:25
	C4	40%		50:50

TOCS: Tea oil camellia shell.

**Table 2:** Physical properties of TOCS-based particleboards.

Group	Treatment	MC (%)	Density (kg/m^3^)	Compaction Ratio
A	A1	9.11 (0.57) *^a^*	700 (10) *^ab^*	0.88 (0.013) *^a^*
	A2	8.91 (0.51) *^a^*	711 (10) *^a^*	0.95 (0.013) *^bc^*
	A3	8.84 (0.49) *^a^*	695 (10) *^abc^*	0.90 (0.013) *^ad^*
	A4	9.36 (0.56) *^a^*	712 (10) *^b^*	0.95 (0.014) *^bc^*
B	B1	9.38 (0.92) *^a^*	694 (14) *^abc^*	0.94 (0.017) *^bd^*
	B2	8.61 (0.45) *^a^*	690 (14) *^abc^*	0.99 (0.020) *^ce^*
	B3	8.39 (0.94) *^a^*	687(10) *^abc^*	0.93 (0.014) *^bd^*
	B4	9.09 (0.75) *^a^*	696 (12) *^abc^*	0.99 (0.017) *^e^*
C	C1	8.97 (0.89) *^a^*	672 (13) *^c^*	0.95 (0.018) *^bc^*
	C2	9.05 (0.78) *^a^*	679 (11) *^ac^*	1.04 (0.017) *^f^*
	C3	9.21 (0.66) *^a^*	676 (12) *^ac^*	0.99 (0.017) *^ce^*
	C4	8.98 (0.43) *^a^*	683 (11) *^ac^*	1.07 (0.018) *^f^*

*abcdef*—values with the same letters are not significantly different by Tukey HSD test (*p* < 0.05).

**Table 3:** Mechanical properties of TOCS-based particleboards.

Group	Treatment	MOR (N/mm^2^)	MOE (N/mm^2^)	IB (N/mm^2^)	FSW (N/mm^2^)	ESW (N/mm^2^)
A	A1	4.50 (1.18) *^a^*	771 (102) *^a^*	0.84 (0.12) *^ab^*	667 (143) *^a^*	501 (78) *^a^*
	A2	5.47 (0.91) *^ab^*	1001 (63) *^b^*	0.81 (0.11) *^ab^*	699 (67) *^abc^*	602 (105) *^ab^*
	A3	4.99 (0.76) *^ab^*	731 (154) *^a^*	0.73 (0.16) *^a^*	718 (162) *^abc^*	590 (83) *^abc^*
	A4	6.48 (0.79) *^bc^*	1098 (124) *^bc^*	0.71 (0.07) *^a^*	738 (155) *^bc^*	653 (86) *^abc^*
B	B1	8.30 (1.59) *^de^*	1117 (163) *^bc^*	0.92 (0.17) *^b^*	698 (80) *^ab^*	516 (64) *^abc^*
	B2	8.74 (1.68) *^de^*	1170 (123) *^bc^*	0.86 (0.10) *^ab^*	715 (99) *^c^*	674 (84) *^abc^*
	B3	8.02 (0.55) *^cd^*	1002 (66) *^b^*	0.78 (0.08) *^ab^*	749 (74) *^abc^*	603 (89) *^abc^*
	B4	8.97 (1.53) *^def^*	1086 (86) *^bc^*	0.74 (0.11) *^ab^*	797 (145) *^abc^*	600 (61) *^abc^*
C	C1	9.71 (1.06) *^defg^*	1196 (171) *^bcd^*	0.81 (0.14) *^ab^*	816 (161) *^abc^*	559 (113) *^abc^*
	C2	10.51 (0.90) *^fg^*	1396 (154) *^de^*	0.72 (0.12) *^b^*	885 (181) *^abc^*	606 (101) *^bc^*
	C3	10.01 (1.32) *^efg^*	1241 (187) *^cde^*	0.75 (0.07) *^ab^*	866 (105) *^abc^*	609 (123) *^abc^*
	C4	11.29 (0.97) *^g^*	1415 (91) *^e^*	0.78 (0.08) *^ab^*	926 (186) *^c^*	684 (54) *^c^*

*abcdefg*—values with the same letters are not significantly different by Tukey HSD test (*p* < 0.05).

**Table 4:** Deminsional stability of TOCS-based particleboards.

Group	Treatment	WA 2 (%)	WA 24 h (%)	TS 2 h (%)	TS 24 h (%)
A	A1	32.0 (10.84) *^ab^*	67.83 (7.39) *^ab^*	6.87 (0.98 *^ab^*	12.87 (0.66) *^ab^*
	A2	29.24 (9.45) *^abc^*	56.72 (9.08) *^acd^*	7.27 (1.04) *^ab^*	12.76 (0.93) *^ac^*
	A3	34.21 (5.63) *^b^*	68.95 (4.73) *^b^*	7.88 (0.75) *^b^*	13.10 (0.76) *^ab^*
	A4	30.75 (6.81) *^abc^*	60.06 (4.44) *^abd^*	7.54 (0.89) *^ab^*	13.38 (0.74) *^b^*
B	B1	22.97 (5.42) *^ac^*	58.36 (3.55) *^ad^*	6.86 (0.55) *^ab^*	12.18 (0.78) *^abcde^*
	B2	23.09 (5.70) *^ac^*	60.41 (8.43) *^abd^*	6.60 (0.55) *^ab^*	12.38 (0.48) *^acde^*
	B3	26.16 (4.94) *^ac^*	60.47 (8.53) *^abd^*	6.82 (1.03) *^ab^*	11.64 (0.99) *^cde^*
	B4	27.31 (8.97) *^ac^*	58.25 (8.71) *^ad^*	6.58 (1.19) *^ab^*	11.25 (0.43) *^cde^*
C	C1	24.70 (12.76) *^ac^*	52.69 (8.67) *^dc^*	6.31 (0.57) *^ab^*	11.92 (0.92) *^abcde^*
	C2	20.13 (6.22) *^ac^*	50.96 (6.33) *^dc^*	6.87 (1.18) *^ab^*	10.93 (0.99) *^de^*
	C3	14.22 (4.90) *^c^*	42.55 (6.59) *^c^*	6.35 (0.37) *^ab^*	10.98 (0.98) *^de^*
	C4	18.63 (5.47) *^ac^*	43.24 (6.30) *^c^*	6.14 (0.69) *^a^*	10.72 (0.65) *^e^*

*abcde*—values with the same letters are not significantly different by Tukey HSD test (*p* < 0.05).

**Table 5:** Thermal conductivity and sound absorption coefficients α of TOCS-based particleboards.

Group	Treatment	Thermal Conductivity (W/mK)	Sound Absorption Coefficients α					
			100 Hz	200 Hz	500 Hz	1000 Hz	1500 Hz	2000 Hz
A	A1	0.1706 (0.007) *^ab^*	0.032	0.057	0.191	0.307	0.324	0.396
	A2	0.1742 (0.008) *^b^*	0.032	0.054	0.198	0.296	0.347	0.374
	A3	0.1507 (0.004) *^cd^*	0.031	0.056	0.184	0.253	0.322	0.374
	A4	0.1549 (0.007) *^cd^*	0.031	0.055	0.202	0.291	0.334	0.355
B	B1	0.1603 (0.016) *^da^*	0.037	0.071	0.196	0.273	0.295	0.289
	B2	0.1606 (0.005) *^da^*	0.032	0.059	0.211	0.323	0.349	0.322
	B3	0.1512 (0.004) *^cd^*	0.036	0.070	0.210	0.265	0.308	0.337
	B4	0.1533 (0.008) *^cd^*	0.035	0.063	0.241	0.299	0.323	0.324
C	C1	0.1440 (0.009) *^c^*	0.035	0.064	0.221	0.295	0.327	0.328
	C2	0.1505 (0.005) *^cd^*	0.035	0.066	0.224	0.286	0.319	0.329
	C3	0.1323 (0.004) *^e^*	0.035	0.064	0.228	0.293	0.323	0.327
	C4	0.1441 (0.009) *^c^*	0.035	0.065	0.224	0.292	0.323	0.328

*abcde*—values with the same letters are not significantly different by Tukey HSD test (*p* < 0.05).

## Data Availability

Not applicable.
